# The Frequency and Causes of Not-Detected Breast Malignancy in Dynamic Contrast-Enhanced MRI

**DOI:** 10.3390/diagnostics12112575

**Published:** 2022-10-24

**Authors:** Donghun Song, Bong Joo Kang, Sung Hun Kim, Jeongmin Lee, Ga Eun Park

**Affiliations:** 1 Department of Radiology, College of Medicine, Bucheon Saint Mary’s Hospital, The Catholic University of Korea, Bucheon-si 14647, Korea; 2 Department of Radiology, College of Medicine, Seoul Saint Mary’s Hospital, The Catholic University of Korea, Seoul 06591, Korea

**Keywords:** breast cancer, dynamic contrast-enhanced MRI, breast MR

## Abstract

Breast MR is the most sensitive imaging modality, but there are cases of malignant tumors that are not detected in MR. This study evaluated the frequency and main causes of malignant breast lesions not detected in dynamic contrast-enhanced (DCE) MR. A total of 1707 cases of preoperative breast MR performed between 2020 and 2021 were included. Three radiologists individually reviewed the DCE MRs and found not-detected malignancy cases in the MRs. The final cases were decided through consensus. For the selected cases, images other than DCE MRIs, such as mammography, ultrasounds, diffusion-weighted MRs, and, if possible, contrast-enhanced chest CTs, were analyzed. In the final sample, 12 cases were not detected in DCE MR, and the frequency was 0.7% (12/1707). Six cases were not detected due to known non-enhancing histologic features. In four cases, tumors were located in the breast periphery and showed no enhancement in MR. In the remaining two cases, malignant lesions were not identified due to underlying marked levels of BPE. The frequency of not-detected malignancy in DCE MR is rare. Knowing the causes of each case and correlating it with other imaging modalities could be helpful in the diagnosis of breast malignancy in DCE MR.

## 1. Introduction

Breast MR has the highest sensitivity for breast cancer detection among several imaging modalities. For this reason, it is widely used for tumor staging and neoadjuvant chemotherapy response assessment and is a recommended method for screening high-risk women [1,2]. Contrast-enhanced MR shows increased signal intensity in T1 weighted images (T1WI) by reducing T1 relaxation time, as the intravenous injected macromolecular contrast agent is extravasated depending on vascular permeability. In the case of a malignant lesion, the formation of leaky vessels during neovascularization increased microvessel permeability, which shows more intense contrast enhancement than the surrounding parenchyma, making it possible to detect it in MR [3,4]. Dynamic contrast-enhanced (DCE) MR shows how the enhancement of the lesion changes with time after contrast media injection—that is, 4-dimensional data, including temporal acquisition, helps in the detection and characterization of lesions [5].

Despite breast MR having the highest sensitivity levels, false negative cases are not found in MR, though they are found in other imaging modalities. The causes include perceptive errors, where the radiologist did not detect the case at the time of the reading; interpretation errors, where the cases are recognized but mistaken for benign lesions; and various technical errors [6]. Previous studies have reported these cases and tried to analyze the causes. Their methodology differs slightly from study to study. Still, overall, the number of malignancies enrolled in these studies has been relatively small, and previous MR scanners were applied [5,7,8,9,10,11,12,13]. Therefore, this study aimed to investigate the frequency and main causes of not-detected breast malignancy in DCE-MR and to find case-by-case solutions.

## 2. Materials and Methods

### 2.1. Study Population

This study was approved by our Institutional Review Board, and informed consent was waived due to its retrospective nature. A total of 2107 preoperative breast MRs with dynamic contrast enhancement (DCE), performed between January 2020 and December 2021, were included in the study. We excluded 362 exams conducted as FUs after neoadjuvant chemotherapy and 38 exams performed after surgical excision or vacuum-associated breast biopsy (VABB). Finally, 1707 cases were enrolled in the study.

### 2.2. Case Selection

For the enrolled 1707 breast MR exams, three radiologists individually sorted out MR not-detected malignancies through report searches and non-blind reviews of DCE MRIs. At this time, reviewers evaluated only DCE MRIs (precontrast and dynamic contrast-enhanced T1WI) of correlating sites while knowing the size and location of the lesion. In this process, 23 cases were selected; 11 retrospectively detected cases, based on consensus between three radiologists, were excluded. Finally, 12 cases with the absence of perceptible enhancement or poor lesion conspicuity at the correlating site were analyzed.

### 2.3. Image Analysis

Three radiologists also reviewed other imaging modalities for 12 lesions that were not visible in DCE MR. First, lesion visibility and signal intensity in the T2WI of the lesion were evaluated. Then, lesion visibility and diffusion restriction were evaluated using diffusion-weighted imaging (DWI) with a high b value of 1000 s/mm^2^ and an apparent diffusion coefficient (ADC) map. In the case of a lesion with diffusion restriction, one radiologist drew an ROI on the lesion on the ADC map and measured the average ADC value. One radiologist evaluated the background parenchymal enhancement (BPE) level in a sequence corresponding to 90 s after contrast media injection according to the BI-RADS 5th edition. Three radiologists evaluated the visibility and imaging features in the mammography or ultrasound performed at the nearest time to the MR exam. If available, the contrast-enhanced chest CT for preoperative staging performed at the nearest time was also analyzed. Based on the image analysis and histology result, the main cause of the not-detected lesions in MR was analyzed and classified.

### 2.4. Histopathology Review

The final histological results from the surgical excisions of 1707 cases were reviewed. For 12 cases not detected in MR, the pathologic reports were referred to for tumor histology, size, location, lymph node involvement, and immunohistochemistry (IHC) type. IHC type was classified based on the following criteria: luminal A (ER and/or PR positive, HER2 negative, and less than 20% of Ki-67), luminal B (ER and/or PR positive, and more than 20% of Ki-67), HER2-enriched (HER2 overexpressed or amplified, ER and PR negative), and triple-negative (ER, PR, and HER2 negative).

### 2.5. MR Protocol

Breast MRs were performed on three different 3T MR machines from two vendors (Verio, Siemens Healthcare, Erlangen, Germany; Ingenia, Philips Medical Systems, Best, The Netherlands). All breast MRs were performed in the prone position with a dedicated breast surface coil. All enrolled patients underwent axial T2-weighted imaging, diffusion-weighted images, and T1-weighted dynamic contrast-enhancement imaging. Detailed protocols are as follows: 

Verio, Siemens Healthcare; (1) Axial, turbo spin-echo T2-weighted-imaging sequence with a TR/TE of 4530/93, a flip angle of 80°, 34 slices, a 320 mm field of view, a matrix size of 576 × 403, 1 excitation, a 4 mm slice thickness, and an acquisition time of 2 min 28 s; (2) axial diffusion-weighted imaging with a readout-segmented echoplanar image (b values 0 and 1000 s/mm^2^) with a TR/TE of 5200/53 ms, respectively; field of view, 340 × 205 mm^2^; matrix size, 192 × 116; slice thickness, 4 mm; acquisition time, 2 min 31 s with 5 readout segments. ADC maps were calculated automatically using software; (3) pre- and post-contrast axial T1-weighted 3D volumetric interpolated brain examination (VIBE) sequences with a TR/TE of 2.7/0.8, a flip angle of 10°, and a slice thickness of 1.2 mm. The images were obtained before and at 10, 70, 130, 190, 250, and 310 s after the injection of gadolinium DTPA (0.1 mmol/kg Gadovist; Bayer Schering Pharma, Berlin, Germany).

Vida, Siemens Healthcare; (1) Axial, turbo spin-echo DIXON T2-weighted-imaging sequence with a TR/TE of 5000/96 ms, a flip angle of 120°, 50 slices, a 320 mm field of view, a matrix size of 448 × 314, a 3 mm slice thickness, and an acquisition time of 3 min 23 s; (2) axial diffusion-weighted imaging with readout-segmented long variable echo trains (b values 0 and 1000 s/mm^2^) with a TR/TE of 4720/60 ms, respectively; field of view, 350× 210 mm^2^; matrix size, 256 × 154; slice thickness of 3 mm; acquisition time of 3 min 29 s with 9 readout segments. ADC maps were calculated automatically using software; (3) pre- and post-contrast axial T1-weighted 3D fast low angle shot (FLASH) sequences with a TR/TE of 4.7/2.27 ms, a flip angle of 10°, and a slice thickness of 1 mm. The images were obtained before and at 10, 93, 176, 259, 342, and 425 s after the injection of gadolinium DTPA (0.1 mmol/kg Gadovist; Bayer Schering Pharma, Berlin, Germany).

Ingenia, Philips Medical Systems; (1) Axial turbo spin-echo T2-weighted imaging with a TR/TE of 3919/80 ms, a flip angle of 90°, a field of view of 300 × 300 mm2, a matrix size of 484 × 300, 2 excitations, a slice thickness of 2 mm, and an acquisition time of 3 min; (2) axial diffusion-weighted imaging with a single-shot spin-echo echoplanar image pulse sequence (b values 0 and 1000 s/mm^2^) with a TR/TE of 12,043.5/102.3 ms, a flip angle of 90°, a FOV of 320 × 320 mm^2^, a matrix size of 184 × 184, and a slice thickness of 3 mm. The ADC map was calculated with a mono-exponential fit using a b value of 1000; (3) pre- and post-contrast T1-weighted high-resolution isotropic volume examination (THRIVE) with a TR/TE of 4.0/1.8 ms, a field of view of 300 × 300 mm2, a matrix size of 332 × 332, 1 excitation, a slice thickness of 1 mm, and a flip angle of 12°. The images were obtained before and at 85, 155, 225, 295, and 365 s after the injection of gadolinium DTPA (0.1 mmol/kg Gadovist; Bayer Schering Pharma, Berlin, Germany).

## 3. Results

The frequency of not-detected lesions in DCE MR was rare at 0.7% (12/1707). Table 1 shows the final histology and frequency of the 1707 cases included in this study. The final histology and frequency of 12 cases were as follows; invasive ductal carcinoma (IDC) (4/1253, 0.3%), invasive lobular carcinoma (ILC) (3/58, 5.2%), mucinous carcinoma (3/50, 6%), ductal carcinoma in situ (DCIS) (1 /233, 0.4%), and metaplastic carcinoma (1/10, 10%).

**Figure 1:**
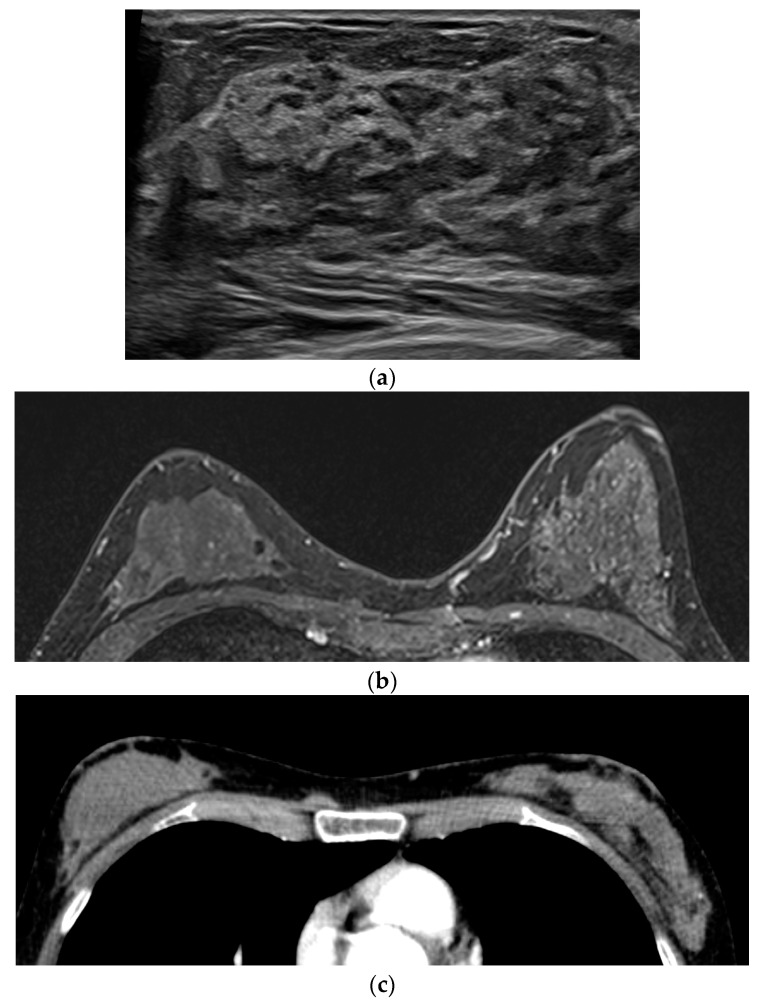
A 42-year-old woman with screening. (**a**) In the ultrasound, there was a 5.5 cm extent diffuse nonmass lesion at the right upper breast, and this lesion was confirmed as an invasive lobular carcinoma on core needle biopsy. (**b**) No discernible enhancing lesion at the right upper breast in the early phase of dynamic contrast-enhanced T1WI. (**c**) Also, no detectable enhancement at the correlating site in the contrast-enhanced chest CT.

Table 2 shows the imaging features and histology of 12 cases of not-detected malignancy in DCE MR and their main causes. Of these cases, 10 were detected by ultrasound and 5 by mammography. Six lesions were only seen by ultrasound and one lesion by mammography. In five cases (Nos. 1–5), lesions could not be distinguished because there was no or little enhancement due to non-enhancing histologic features; mucinous carcinoma (n = 3), ILC (n = 2) (Figure 1). 

**Figure 2:**
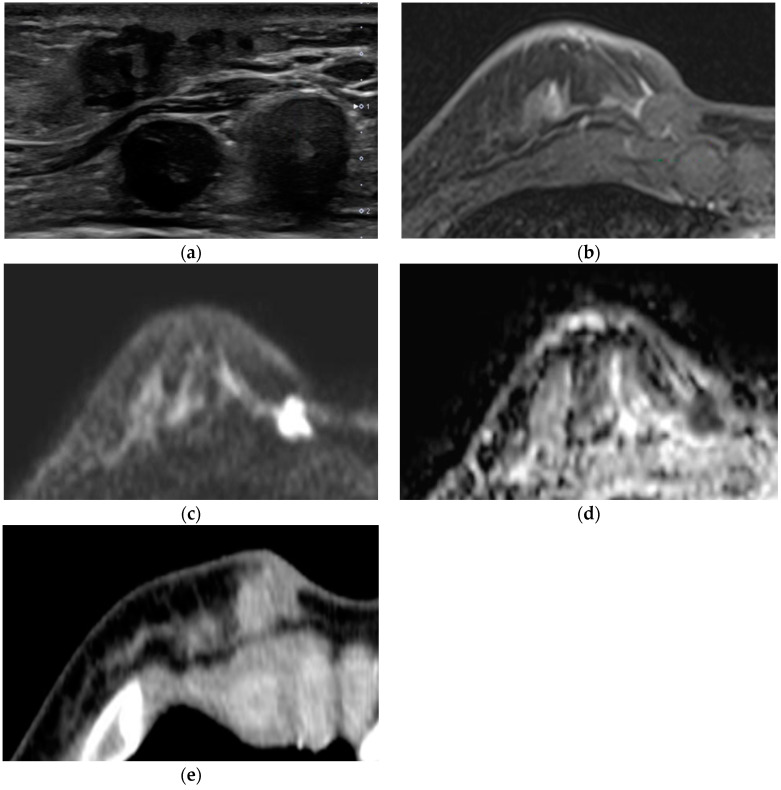
A 42-year-old woman with palpable mass at breast periphery. (**a**) In the ultrasound, a 2.3 cm irregular hypoechoic mass was detected in the palpable site of the right breast at the 4-o’ clock periphery and confirmed as invasive ductal carcinoma by core needle biopsy. (**b**) No discernible enhancing lesion at the correlating site in the early phase of dynamic contrast-enhanced T1WI. This palpable mass showed definite diffusion restriction with an ADC value of 0.68 × 10^−3^ in DWI b = 1000 (**c**), ADC map (**d**), and homogeneous enhancement in contrast-enhanced chest CT in the supine position (**e**).

In four cases (Nos. 6–9), the lesions were located in the breast periphery or chest wall and showed no enhancement; IDC (n = 3), metaplastic carcinoma (n = 1). These lesions were not detected in DCE MR, but all showed definite diffusion restriction, and the measured ADC values ranged from 0.68 to 0.96 × 10^−3^ mm^2^/s. Three of these lesions showed distinct homogeneous enhancement on contrast-enhanced chest CT (Nos. 6, 8, and 9) (Figure 2).

**Figure 3:**
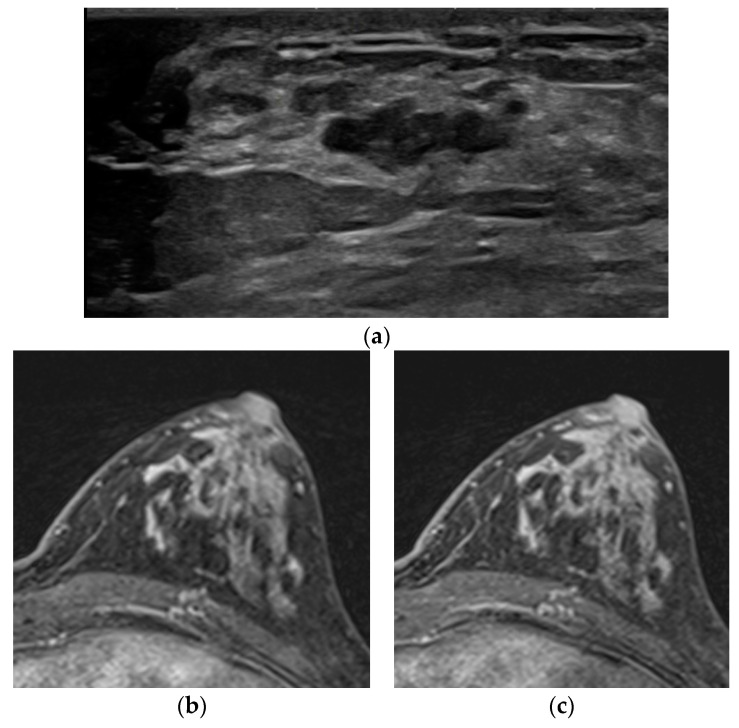
A 53-year-old woman with screening. (**a**) In the ultrasound, a 2 cm irregular hypoechoic mass was detected in the left breast in the 3-o’ clock direction and confirmed as invasive ductal carcinoma with ductal carcinoma in situ. The final pathology revealed 0.5 cm-sized invasive cancer with 2.4 cm-sized DCIS. No discernible lesion was identified in the subtraction image from the early phase (**b**) or the delayed phase of T1WI due to the marked level of BPE (**c**).

The other case was a patient who complained of a growing palpable mass at the far upper breast with a history of injection mammoplasty. At first, there was no enhancing lesion at the palpable site, but there was a mass with definite diffusion restriction. The MR re-examination was performed with the prone position considering the location of the palpable site, and an irregular mass with heterogeneous enhancement was confirmed (No. 7). In three cases (Nos. 10–12), lesions could not be distinguished in DCE MR due to marked levels of BPE. Further, lesions were not distinguished in both T2WI and DWI but were identified in ultrasound or mammography (Figure 3).

## 4. Discussion

In this study, the frequency of not-detected malignancy in MR was 0.7% (12/1707). This study included consecutive preoperative MR for two years, and almost all breast cancer patients underwent preoperative MR in this hospital. In several published studies on MR not-detected malignancy, the false negative rate was about 5~15% [5,7,8,9,10,11,12]. Our study showed a remarkably low rate compared to previous studies. However, caution is warranted in directly comparing false-negative rates since each study has different MR indications, inclusion, and exclusion criteria. In particular, the fact that a non-blind review of MR with known malignancy was performed and that retrospectively detected cancer was excluded may also have had an impact. Two previous studies were also conducted based on MR with known malignancy with the retrospective review. The rate of not-detected malignancy was 3.2% (7/222) [8] and 8.7% (9/104) [9], respectively, and the rate was relatively low in the later conducted study [8].

There are also differences from previous research results. In previous studies, the not-detected rate of DCIS was significantly higher than that of invasive cancer. In our result, only one out of 233 DCIS lesions was not detected in MR, and the not-detected rate was also lower than that of invasive cancer. While our study was conducted in 3T MR scanners with a higher spatial resolution (slice thickness 1~1.2 mm) and a higher temporal resolution (60~83 s), 1T or 1.5T MR was used in previous studies. MR scanning protocols have evolved rapidly in a short period. As the resolution and overall quality of MR images have improved, the detectability of lesions has increased. Above all, it would have contributed greatly to the increase in the detection rate of small lesions, especially DCIS, confined in ducts surrounded by normal breast tissue. In addition, as the number of breast MR exams gradually increases, the reading skills of radiologists have likely improved.

We defined and classified three main causes. The first is rare but known as not-enhancing histologic features. Mucinous carcinoma has abundant extracellular mucin secreted by tumor cells and shows high SI in T2WI. DCE MR is known to show various enhancement patterns depending on the distribution of solid and mucinous components; however, when mucin is predominant, there is almost no enhancement in some cases [14,15]. In our cases of mucinous carcinoma, lesions were not identified using DCE MR only but were identified after correlating with T2WI. However, since the T2WI feature of mucinous carcinoma, a well-circumscribed mass with T2 high SI, also overlaps with the benign feature, attention should be paid to MR interpretation [6,16]. ILC had a distinctive histologic feature of an “Indian File” growth pattern that diffusely infiltrates the adjacent breast parenchyma [17]. Perhaps because of this unique growth pattern, some lobular carcinomas might be able to receive a vascular supply through the diffusion of the preexisting parenchymal capillary network, which leads to less dependence on neovascularization [9]. In our cases of ILC, conventional imaging, such as mammography and ultrasound, was more useful in lesion detection.

The second is by location. During the breast MR examination, the patient lies in a prone position, with the chest hanging freely in the recesses of the coil. This method allows breast tissue to spread and prevents respiratory motion artifacts, ultimately facilitating lesion detection [18]. If the lesion is located on the breast periphery or chest wall, contrast media may not be sufficiently delivered due to this posture [6]. In the four cases, no lesions were found in DCE MR. On the other hand, these lesions demonstrated diffusion limitation and low-range ADC values in DWI independent of the contrast agent [19,20]. Three of these lesions showed homogeneousness or rim enhancement in contrast-enhanced chest CTs performed in the supine position. Although the mechanisms of contrast enhancement in CT and MR are different, this indicates that the location does matter.

The last one is the marked level of BPE. BPE is well known as a factor that increases the recall rate and false positive rate in breast MR readings. In particular, if the BPE is at a marked level, it may be almost impossible to distinguish the lesion from the breast parenchyma [21]. In the three cases, lesions were masked by the marked level of BPE in DCE MR; neither T2WI nor DWI was helpful in lesion identification. Conventional imaging, such as mammography or ultrasound, was more valuable. As an approach to minimize the effect of BPE, it is proposed to consider the menstrual cycle [22] or to add an ultrafast sequence [23].

This study has several limitations. First, although a relatively large number of consecutive MR exams were included, this study was conducted by a single institution. This retrospective study was conducted through a non-blind review of MR in patients with known breast cancer for staging. In addition, all 3T MR scanners used in this study had high temporal and spatial resolution. These factors may have underestimated the frequency of not-detected malignancy in DCE MR. Case selection was made through the consensus of three radiologists, but this process may contain a subjective element.

## 5. Conclusions

The frequency of not-detected malignancy in DCE MR was rare (12/1707, 0.7%), which was less than reported in previously published studies. Knowing the causes of each case and correlating it with other imaging modalities could be helpful in the diagnosis of breast malignancy.

## Figures and Tables

**Table 1:** Frequency of not-detected breast malignancy in DCE MR according to pathology.

Pathology	N (% ^*^)	Not-detected case N (% ^†^)
Invasive ductal carcinoma	1253 (73.4%)	4 (0.3%)
Invasive lobular carcinoma	58 (13.7%)	3 (5.2%)
Mucinous carcinoma	50 (3.5%)	3 (6%)
Ductal carcinoma in situ	233 (2.4%)	1 (0.4%)
Metaplastic carcinoma	10 (0.6%)	1 (10%)
Others	103 (5.9%)	-
Total	1707	12 (0.7%)

* % number of each pathology/total number; **^†^** % number of not-detected cases/number of each pathology; N = number, IDC = invasive ductal carcinoma, ILC = invasive lobular carcinoma, DCIS = ductal carcinoma in situ.

**Table 2:** Imaging features and histology of not-detected breast malignancy in DCE MR.

No.	Age	Pathologic Feature		DCE MR		Cause	Other Imaging Features			
		Pathology	Tumor Size (mm)	Location	BPE		T2WI	DWI	MMG	US
1	42	ILC	55	Rt central	minimal	Histologic feature	Diffuse low	-	Global asymmetry	Diffuse nonmass
2	45	ILC	12	Lt 11-o’ clock	minimal	Histologic feature	-	-	Asymmetry	Mass
3	46	mucinous carcinoma	24	Lt 2-o’ clock	moderate	Histologic feature	High	-	-	Mass
4	57	mucinous carcinoma	9	Lt 11-o’ clock periphery	mild	Histologic feature	High	-	-	Mass
5	69	mucinous carcinoma	13	Lt 9-o’ clock	moderate	Histologic feature	High	-	Mass	Mass
6	44	IDC	15	Rt 5-o’clock periphery	minimal	Location	Intermediate	+	-	Mass
7	52	IDC	20	Lt far upper	mild	Location	Intermediate	+	-	-
8	42	IDC	23	Rt 4-o’ clock periphery	moderate	Location	Low	+	-	Mass
9	67	metaplastic carcinoma	17	Lt 1-o’clock, chest wall	minimal	Location	Intermediate to high	+	-	Mass
10	62	ILC	20	Rt 1-o’ clock	marked	BPE	-	-	Focal asymmetry	Mass
11	53	IDC	5	Lt 3-o’ clock	marked	BPE	-	-	-	Mass
12	36	DCIS	45	Lt 12-o’ clock	marked	BPE	-	-	Microcalcifications	-

BPE = background parenchymal enhancement, MMG = mammography, IDC = invasive ductal carcinoma, ILC = invasive lobular carcinoma, DCIS = ductal carcinoma in situ, ADC value = 10^−3^ mm^2^/s.

## Data Availability

All data generated and analyzed during this study are included in this published article. Raw data supporting the findings of this study are available from the corresponding author upon request.
